# Enough is enough: how many rectal suction biopsies do you need to diagnose Hirschsprung’s disease?

**DOI:** 10.1007/s00383-024-05793-y

**Published:** 2024-07-22

**Authors:** Hannah Rachel Neeser, Isabella Robbiani, Ann-Katrin Rodewald, Tobias Nigbur, Anthony di Natale, Ueli Moehrlen, Sasha Job Tharakan

**Affiliations:** 1 Department of Pediatric Surgery, University Children’s Hospital Zurich, Steinwiesstrasse 75, 8032 Zurich, Switzerland; 2 Institute for Pathology and Molecular Pathology, University Hospital Zurich, Schmelzbergstrasse 12, 8091 Zurich, Switzerland; 3 Independent Statistician, Zurich, Switzerland

**Keywords:** Hirschsprung’s disease, Rectal suction biopsies, Infant, Pathology

## Abstract

**Purpose:**

Rectal suction biopsy (RSB) is the gold standard for diagnosing Hirschsprung’s disease (HD) in infants. Despite being a common procedure, no standard exists on the number of biopsy specimens and their respective level within the rectum.

**Methods:**

We conducted a retrospective review of epidemiological and pathological data of patients who underwent RSB at our institution between January 2011 and May 2022. During RSB we obtain 4 specimens: at 1 cm, 3 cm and 5 cm above the dentate line, besides one specimen at the dentate line. We used a logistic regression model for statistical analysis and included control variables (e.g. underlying disease, weight at first biopsy, gestational age).

**Results:**

A total of 92 patients underwent 115 biopsies, with an average of 3.77 specimens per session. Of the specimens taken at 1 cm above the dentate line 73.9% were conclusive, at 3 cm 75.9% and at 5 cm 79.2%. Specimens taken at the dentate line were squamous or transitional epithelia in 31.5% and therefore of no use for HD diagnostics. The specimen at 3 cm shows the highest discriminative power whether the biopsy session was diagnostic (*p*-value < 1%).

**Conclusions:**

We propose that a total of three specimens, namely one at 1 cm, one at 3 cm and one at 5 cm above the dentate line, is enough to diagnose or exclude HD.

## Introduction

Pediatric surgeons frequently encounter neonatal intestinal obstruction and defecation problems. Therefore, ruling out Hirschsprung’s disease (HD) is a common practice. HD is a recognized developmental disorder of the enteric nervous system leading to the absence of ganglion cells in the myenteric and submucosal plexuses of, most commonly, the distal colon. As a result of the failure of migration of the ganglion cells from the neural crest, this aganglionosis causes severe constipation and functional obstruction in neonates.

Rectal suction biopsy (RSB) is considered the gold standard in the diagnostic work-up of HD [1, 2]. However, there is no consensus on many aspects of its current use with significant differences regarding the number and location of specimens routinely taken [3, 4]. Despite the simplicity of the procedure, the need for repeat biopsies is high, estimated to be between 4 and 40% [4–8]. Having a standard approach may help to minimize the number of repeat biopsies and thereby reduce patient effort and healthcare costs.

Within our institution, a standard approach to performing RSB was implemented more than 15 years ago and is employed by all surgeons, if possible. We strive to take a total of four specimens per biopsy session. These are taken at the dentate line, 1 cm, 3 cm and 5 cm above the dentate line. The rationale for our most distal specimen, taken directly at the dentate line, has historically evolved at our institution to rule out ultra-short HD, a rare form of HD affecting the most distal rectum [9, 10]. Following consultation with our pathology department, the location and number of the other three specimens were agreed upon. In light of the recent literature [3, 4] indicating significant variation in the execution of RSB across institutions and our doubt regarding the significance of the biopsy at the dentate line, we sought to evaluate our RSB practice.

Therefore, the aim of this study is to determine the diagnostic value of different specimen locations within the rectum and to thereby establish the minimum number of rectal suction biopsies required to diagnose or rule out HD.

## Methods

### Data collection

This retrospective study was conducted at our institution after receiving approval from the local ethics committee (BASEC 2022-01007). Patients who received at least one RSB to diagnose or exclude HD between January 2011 and May 2022 and who had documented consent were included. We retrieved the following data from the patients’ medical records: demographic variables, clinical presentation and work-up with particular attention to the RSB sessions including indication, sample characteristics and histopathological work-up. Archived biopsies were reviewed by a certified pathologist if the original pathology report only summarized the results of all biopsies, and thus prevented detailed data extraction on the individual samples. No new tests were performed on the samples.

### Suction rectal biopsy technique

**Fig. 1:**
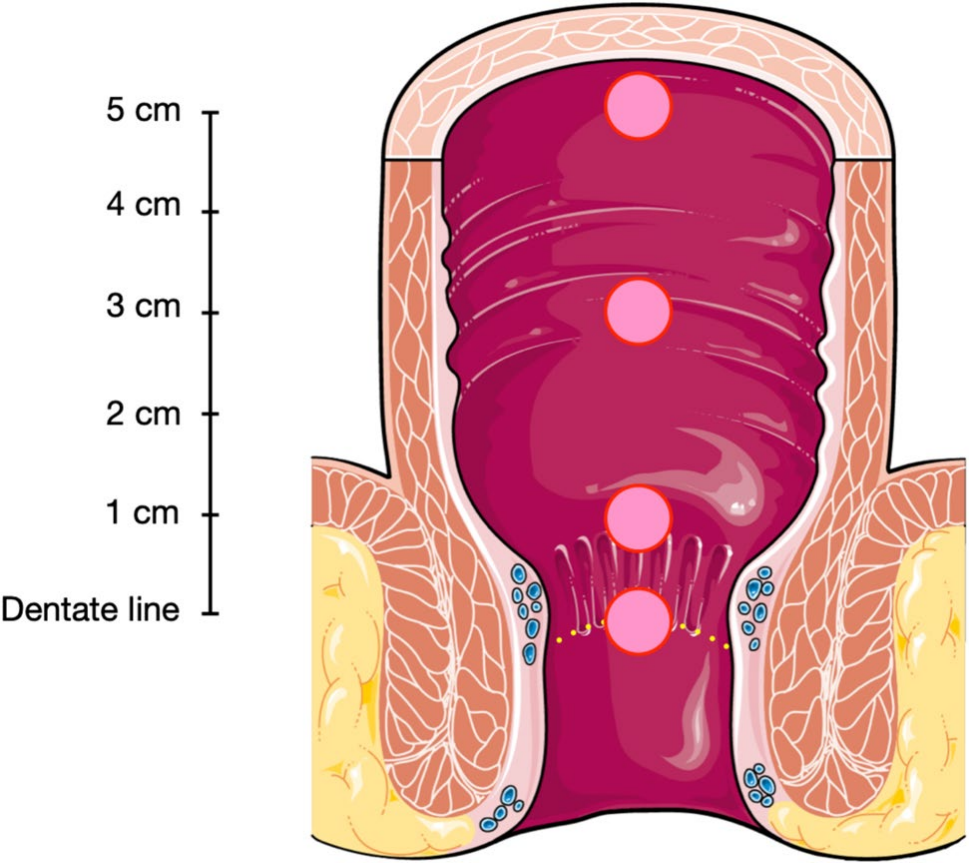
Locations within rectum for RSB. Dotted yellow line: Dentate line. Pink circles: biopsy locations. (Figure modified with text, markings, and annotation after adaptation of “Rectum” from Servier Medical Art by Servier, licensed under a Creative Commons Attribution 3.0 Unported License)

We perform RSB on all infants under 6 months, and depending on the case, up to the age of 18 months to rule out HD. We perform the biopsies without sedation or anesthesia. The set utilized is the rbi2 Suction Rectal Biopsy System (Aus Systems^™^) with a 10 ml syringe to generate suction. The patient is supine, and the feet are held upwards by an assistant. We strive to take one specimen at 5 cm, one at 3 cm and one at 1 cm above the dentate line, as well as one specimen at the dentate line. Further, all specimens are obtained from the dorsal rectal wall as illustrated in Fig. 1. The depth measuring indicators on the capsules are employed to ascertain the location within the rectum.

### Histopathological work-up

**Fig. 2:**
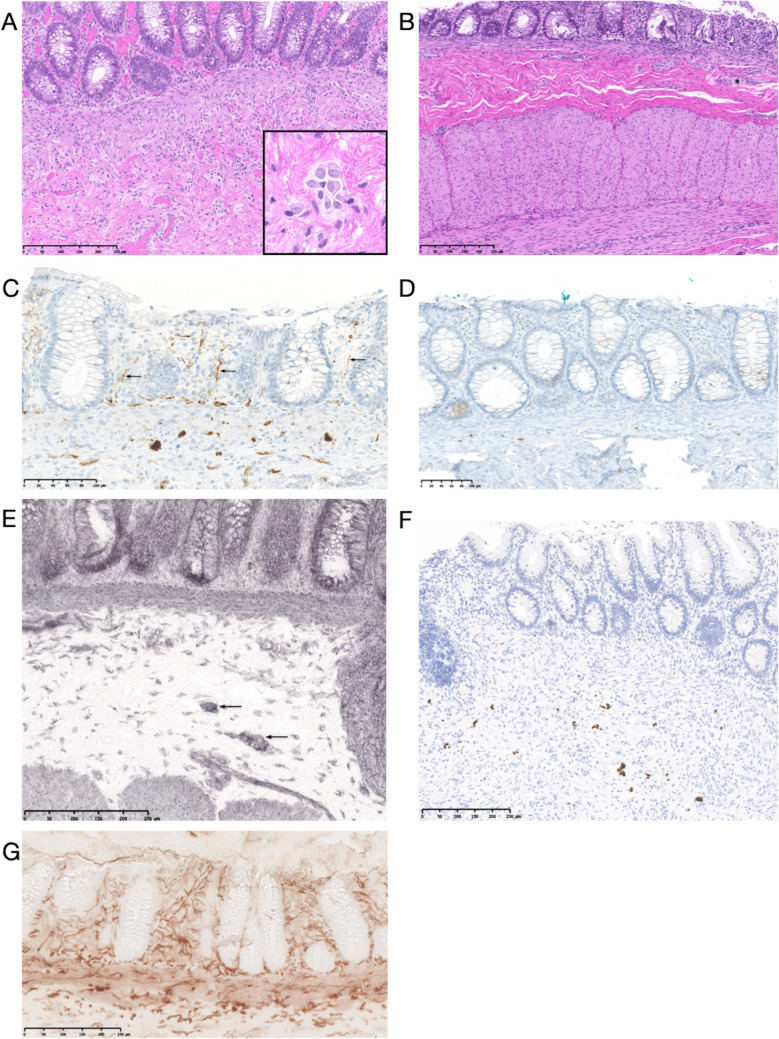
**A** Normal rectal mucosal biopsy with evidence of submucosal ganglion cells (hematoxylin–eosin stain, H&E) Inset: group of regular ganglion cells. **B** Rectal mucosal biopsy without detectable ganglion cells (H&E). Note the submucosal and myenteric nerve fibers (asterisks), which may be hypertrophic in HD. **C** Typical ganglionic rectal biopsy, many calretinin immunoreactive mucosal fibers are stained (arrows). **D** Typical aganglionic rectal biopsy, calretinin immunoreactive mucosal fibers are completely absent; normally distributed mast cells (weak nuclear and cytoplasmic brown staining) serve as an internal control. **E** Lactate dehydrogenase enzyme histochemistry of frozen section biopsy highlighting clusters of submucosal ganglion cells (arrows). **F** Nuclear positive Phox2b immunoreactivity in ganglionic mucosa biopsy highlights neuronal cell bodies (ganglion cells). **G** Acetylcholinesterase enzyme histochemistry of frozen section biopsy in HD: visualization of strong and branched fibers as a sign of increased cholinergic innervation in lamina propria and muscularis mucosa

Pathologic evaluation of RSB involves two approaches. The first uses paraffin serial sections stained with hematoxylin and eosin (H&E). In questionable cases where diagnosis is not possible with H&E alone, we additionally perform Calretinin and/or Phox2b immunohistochemistry to substantiate the diagnosis. Secondly, enzyme histochemistry on frozen sections is employed to identify ganglion cells in submucosal layers (L-lactatdehydrogenase, LDH) and acetylcholinesterase (AchE)-positive nerves in mucosal lamina propria. As we typically have four samples from different levels to evaluate, at least one sample is subjected to enzyme-histochemical assessment. Figure 2 depicts the various stains we utilize.

For the purpose of this study, a conclusive sample was defined as in Table 1. If the sample did not fulfill any of the four described combinations it was marked as non-conclusive. The pathologist responsible for the case defined the submucosal tissue as “sufficient” if it measured at least a third as much as the mucosal tissue.

**Table 1:** Definition of conclusive samples

	Conclusive normal finding	Conclusive for HD
Paraffin serial sections		
In hematoxylin and eosin stain	Visible ganglion cells	Absence of ganglion cells
	AND	AND
Immunohistochemistry	Positive fibers with Calretinin OR ganglion cells in Phox2b	Negative fibers with Calretinin OR absence of ganglion cells in Phox2b
Frozen sections		
Enzyme-histochemistry	Normal fiber activity with AchE AND visible ganglion cells with LDH	Increased AchE activity AND absence of ganglion cells with LDH

*HD* Hirschsprung’s disease

### Statistical analysis

Statistical analysis was performed using R Statistical Software (4.2.1; R Core Team 2023). For the descriptive statistics, we included all RSB samples and all biopsy-sessions. To estimate the likelihood of overall conclusiveness of a biopsy session based on the conclusiveness of the samples taken at different locations we use a logistic regression. For the logistic regression alone, we exclude specimens taken at the dentate line due to the frequent contamination with squamous/transitional epithelia, as well as biopsies taken at locations other than 1, 3 and 5 cm. We also restrict the sample to first biopsies to avoid potential biases and to biopsy sessions including all three locations. To exclude confounding effects, we include a set of control variables in a second logistic regression: gender, underlying condition, weight at the time of first biopsy above 4.5 kg, and as continuous variables gestational age (weeks, de-meaned) and age at biopsy (weeks, de-meaned). For these logistic regression models, we report *p*-values for variable significance. To check for multicollinearity, we report the maximum variance inflation factor (VIF) among all variables, where values above 5 would be deemed problematic. The area under the curve (AUC) is reported for correct classification of the prediction, where a value of 0.5 would arise for random classification and 1.0 for perfect classification. Finally, the Hosmer–Lemeshow Test *p*-value is reported as a measure of the goodness of fit for the respective model. A value below 0.1 indicates a poor fit.

## Results

### Patient characteristics

On chart review, we found 119 patients who had undergone RSB at our institution between January 2011 and May 2022. We received consent from 92 patients’ families to include these patients’ data in our study.

Sixty-two patients (67.4%) were male, and the mean gestational age was 37.82 weeks, whereas 15 patients (16.3%) were born prematurely. Around one-third of our patients (*n* = 25, 27.2%) had an underlying condition, the most common being Trisomy 21 (*n* = 6, 6.5%) and/or congenital heart defects (*n* = 6, 6.5%). The first biopsy session was performed at a median age of five weeks and a median weight of 3.96 kg. The most common indication for RSB overall was the presence of a neonatal bowel obstruction (*n* = 57). The most common indication for biopsies in patients with negative biopsies for HD were defecation problems (*n* = 25; 44.6% of non-HD patients). The final diagnosis was confirmed to be HD in 36 (39.1%) patients. In 54 (58.7%) patients, HD could be ruled out and in two patients the biopsy results, despite repeat biopsies, were not conclusive, but long-term follow-up in both patients showed no further clinical evidence of HD. Two patients (2.2%) had a complication following suction rectal biopsies: both patients had ongoing rectal bleeding postoperatively necessitating an operative procedure to achieve hemostasis. More detailed patient demographics are shown in Table 2.

**Table 2:** Patient characteristics

(*n*)	All patients (92)	HD positive (36; 39.1%)	HD negative (56; 60.2%)
Gestational age (mean weeks, ± SD), thereof	37.82 (± 3.87)	38.39 (± 2.74)	36.8 (± 4.42)
Term born (≥ 37 0/7)	77 (83.7%)	33 (91.7%)	44 (78.6%)
Preterm (≤ 36 6/7)	15 (16.3%)	3 (8.3%)	12 (21.4%)
Birthweight (mean kg, ± SD; median)	3.05 (± 0.93, 3.28)	3.05 (± 0.94; 3.26)	3.05 (± 0.94; 3.28)
Gender Male	62 (67.4%)	28 (77.8%)	34 (60.7%)
Underlying condition	25 (27.2%)	7 (19.4%)	18 (32.1%)
Age at first RSB (mean weeks ± SD; median)	12.4 (± 20.64; 5)	10.33 (± 30.08, 2)	13.46 (± 11.20; 10)
Weight at first RSB (mean kg, ± SD; median)	4.65 (± 2.15; 3.96)	4.00 (± 2.09; 3.38)	5.06 (± 2.10;4.29)
Mean number of RSB sessions (± SD)	1.29 (± 0.58)	1.36 (± 0.59)	1.25 (± 0.58)
Patients needing full-thickness biopsies, *n* (%)	4 (4.3%)	2 (5.5%)	2 (3.6%)
Complications from RSB, *n* (%)	2 (2.2%)	0	2 (3.6%)

*HD* Hirschsprung’s disease, *SD* Standard deviation

### Rectal suction biopsies

We performed a minimum of one RSB session on 92 patients during the study period. Most patients (*n* = 71, 77.2%) required one biopsy session for a conclusive biopsy result. In 15 patients (16.3%) a second and in 6 patients (6.5%) a third session had to be performed before a conclusive result was found. Two of the second and two of the third sessions were full-thickness biopsies and are not included in our analysis. Thus, we analyzed a total of 115 RSB sessions with a mean of 3.77 specimens per session. Details of the biopsy results are shown in Table 3.

**Table 3:** Biopsy results

Specimen location	At dentate line	1 cm above dentate line	3 cm above dentate line	5 cm above dentate line	Other locations^a^
Total number of biopsies analyzed	92	115	109	101	14
Mean diameter in mm (± SD)	3.14 (± 0.98)	2.89 (± 0.1.07)	3.70 (± 0.1.45)	3.50 (± 0.1.32)	3.46 (± 1.05)
Number of biopsies with insufficient submucosa (%)	26 (28.3%)	14 (12.2%)	14 (12.8%)	16 (15.8%)	0
Number of conclusive probes (%)	45 (48.9%)	85 (73.9%)	82 (75.2%)	80 (79.2%)	11 (78.6%)

*HD* Hirschsprung’s disease, *SD* Standard deviation

^a^Other locations were 9 specimens at 2 cm and 5 specimens at 4 cm above the dentate line

The mean size of the samples-measured during pathological work-up-was similar in all locations, apart from the 1 cm location, which were slightly smaller. However, the standard deviations for each location indicate no statistically significant differences. In addition, we did not find any indication that biopsy sizes depend on age at first consultation. In all locations-excluding the dentate line samples-most of the specimens yielded enough submucosa for histopathological analysis (around 85%). At the dentate line location almost a third of biopsies (28.3%) showed insufficient submucosal tissue.

The specimens from the dentate line were conclusive in 48.9%. The main reason for the high rate of inconclusive biopsies was that 31.5% of these samples (*n* = 29) were shown to be squamous or transitional epithelium on pathological examination and could therefore not be used for HD diagnostics. At 1 cm 73.9% of specimens were conclusive, at 3 cm 75.9% and at 5 cm 79.2%.

In univariate logistic models we find the 3 cm location to have the highest odds for overall conclusiveness. It exhibits a high significance level and has the highest discriminative power among all three locations. In the further logistic models, we add the previously mentioned control variables, and the results are similar to the univariate models. The models’ discriminative power, as measured by the area under the curve (AUC), is the highest for the 3 cm location with an AUC value of 0.76. Regarding the control variables, only the indicator variable indicating whether the child has a weight above 4.5 kg is robustly significant, indicating that children with a higher weight have a higher likelihood of being overall conclusive. The detailed results of our logistic regressions, as described in the Methods section, can be found in Table 4.

**Table 4:** Detailed results of the logistic regressions

Intercept	− 0.18	− 0.92^a^	− 1.10^a^	− 2.57^c^	− 0.50	− 2.12^b^	− 0.82	− 4.44^b^
*p*-value	*0.67*	*0.06*	*0.06*	< *0.01*	*0.45*	*0.05*	*0.24*	*0.01*
1 cm specimen	2.09^c^			1.30^a^	2.44^c^			2.22^b^
*p*-value	< *0.01*			*0.08*	< *0.01*			*0.02*
3 cm specimen		3.37^c^		2.39^c^		4.54^c^		3.96^c^
*p*-value		< *0.01*		< *0.01*		< *0.01*		< *0.01*
5 cm specimen			3.11^c^	1.96^b^			3.02^c^	1.65^a^
*p*-value			< *0.01*	*0.02*			< *0.01*	*0.10*
Gender					0.18	− 0.37	− 0.16	− 0.77
*p*-value					*0.79*	*0.64*	*0.81*	*0.40*
Underlying condition					− 1.20	− 0.84	− 1.23	− 0.52
*p*-value					*0.14*	*0.41*	*0.16*	*0.63*
Weight at first biopsy					1.56^b^	2.70^b^	0.55	3.12^b^
*p*-value					*0.05*	*0.03*	*0.48*	*0.03*
Gestational age					− 0.18	− 0.07	− 0.12	− 0.07
*p*-value					*0.12*	*0.58*	*0.28*	*0.62*
Age at biopsy					− 0.03^a^	− 0.01	− 0.01	− 0.02
*p*-value					*0.09*	*0.45*	*0.71*	*0.41*
Max. VIF	–	–	–	1.05	1.56	2.4	1.59	3.05
AUC	0.59	0.71	0.67	0.75	0.65	0.76	0.68	0.79
HL *p*-value	–	–	–	0.70	0.54	0.66	0.61	0.89
Observations	84	84	84	84	84	84	84	84

The *p*-values in italics refer to the non-italic value in the row above

The variables are defined as follows: for the biopsy locations as 1 if the respective location was conclusive and 0 if otherwise. Gender is 1 for female and 0 for male. Underlying condition 1 for present and 0 for not present. Weight at first biopsy is 1 for patients weighing over 4.5 kg and 0 if they weighed less. The continuous variables gestational age (weeks) and age at biopsy (weeks) are de-meaned using the median

*AUC* Area under the curve, *HL* Hosmer–Lemeshow-Test, *VIF* variance inflation factor

Based on the variable *p*-values we denote significance level 1% with^c^, 5% with^b^ and 10% with^a^

We found no conclusive evidence in our cohort of the existence of ultra-short HD as defined by Bruder et al. [9]. It could, however, not be ruled out in the initial biopsy session on pathological examination in 3 patients. In two of these, a repeat biopsy showed ganglion cells at 1 cm, so that ultra-short HD could be excluded. In one patient a further biopsy was not performed, however, clinical follow-up over several years gave no further indication for an ultra-short HD.

## Discussion

To the best of our knowledge, this retrospective study is the first to demonstrate that the biopsy location within the rectum may impact its diagnostic usefulness, thereby influencing the decision regarding which locations to biopsy. We were able to demonstrate that if the 3 cm specimen is conclusive, the probability of the entire biopsy session being conclusive is significantly higher compared to other levels.

A single biopsy specimen obtained during RSB is insufficient for the diagnosis or exclusion of HD for several reasons. With appropriate technical execution, it is possible to obtain satisfactory specimens using RSB. The individual specimens should be of an adequate size, approximately 2–3 mm, and should have sufficient submucosal tissue attached to them [11, 12]. In our cohort, RSB frequently yields biopsies of an adequate size, often with sufficient submucosal tissue. Nevertheless, obtaining more than one specimen is prudent, as one out of four specimens is likely to be inconclusive.

Furthermore, the distal rectum exhibits a physiological hypoganglionosis of up to 2 cm proximal to the dentate line, which may be difficult to differentiate from HD for pathologists inexperienced in HD diagnosis [13, 14]. Thus, at least one specimen should be taken at 3 to 5 cm above the dentate line to ensure that the sampled area is outside of the physiologically hypoganglionic zone. Biopsies taken at the most distal locations can also be squamous epithelia of the anal canal or transitional epithelia, which can extend up to a few millimeters above the dentate line. This was evidenced by our cohort, where up to a third of our most distal biopsies taken at the dentate line were found to be squamous epithelia. These specimens are not useful for diagnosing HD [13, 15] and therefore we will not continue to obtain these specimens.

To ensure that a possible ultra-short HD is not missed, clinicians are often counselled to take a “low” biopsy at approximately 1 cm above the dentate line [11, 12]. This subtype of HD is thought to be limited to the distal 3–4 cm of the rectum and affects up to 5% to over 10% of all HD patients [9, 16]. However, in the literature the definition is not uniform [12]. As discussed above the distal rectum is hypoganglionic and, therefore, the diagnosis of ultra-short HD is not straightforward. It relies heavily on enzyme histochemistry, which requires an extra, frozen biopsy [16, 17].

In addition, we know that the transition zone in HD from the pathological aganglionic zone to the euganglionic zone can stretch over several centimeters. Consequently, it is possible that only partial circumferential hypo- or aganglionic segments may be identified, which could potentially lead to misinterpretation [18]. As specimens are typically obtained from the dorsal rectal wall, it is prudent to obtain more than just one biopsy proximally to ensure that the full extent of the disease has been captured.

These arguments alone mean that at least two or three biopsies would be necessary to safely rule out HD. In addition, pathologists prefer to have more than one biopsy for different examination methods. The two most common approaches to diagnosing HD are by demonstrating the absence of ganglion cells in the submucosal plexus in H&E stained paraffin serial sections or by demonstrating increased fiber activity with acetylcholinesterase using enzyme histochemistry on frozen sections [3, 4]. Our pathology department also uses Calretinin and Phox2b-immunohistochemistry, as well as L-lactatdehydrogenase enzyme histochemistry. As other authors have noted, performing all these tests at several different levels would undoubtedly allow the most accurate diagnosis to be made. However, considering resources and the invasiveness of obtaining the material, a compromise must be made [11]. It is crucial for clinicians and pathologists to cooperate closely to achieve the most favorable outcomes that are realistic under the circumstances and constraints of each institution.

Despite highlighting some important facts, our study’s retrospective nature introduces inherent limitations. The study’s objective is based on the absence of a standard approach for obtaining RSB, necessitating the design of a single-center study. This approach is associated with some drawbacks, including a small cohort. Further, as a national center for referral of patients with suspected HD, we may have introduced a selection bias towards patients with underlying congenital conditions.

Based on our data, we suggest that a specimen at 3 cm from the dentate line should be taken in all biopsy sessions since it has the highest odds for overall conclusiveness. Given the possibility of ultra-short HD, we conclude that we should continue to take a biopsy at 1 cm from the dentate line. Due to potential non-linear transition zones, we further propose obtaining a third biopsy. There are several potential locations for the third biopsy. To rule out non-linear transition zones, an additional biopsy at 3 cm (e.g. 3 or 9 o’clock in the lithotomy position) may be advisable. To increase diagnostic information of potential ultra-short HD a third biopsy at 5 cm is valuable: visible ganglion cells at 5 cm with absent ones more distally mean an ultra-short HD may be a possibility. At our institution, we will continue to take the third biopsy at 5 cm.

We believe that having a standard approach, as outlined above, may not only reduce the necessity for repeat biopsies but also assist in the understanding of HD and its variants by obtaining optimal biopsy results. This becomes even more important as pathological HD diagnostics are improving and new methods are being developed as we are continuing to learn about HD and its possible variations [17].

## Conclusion

We conclude that 3 biopsies are enough to diagnose or rule out HD. We suggest taking biopsies at 1 cm, 3 cm and 5 cm above the dentate line. A biopsy at the dentate line is not necessary.
